# Differential Proliferative Characteristics of Alveolar Fibroblasts in Interstitial Lung Diseases: Regulative Role of IL-1 and PGE_2_

**DOI:** 10.1155/S0962935194000633

**Published:** 1994

**Authors:** Elizabeth Fireman, Shlomo Ben Efraim, Joel Greif, Hava Peretz, Shmuel Kivity, Marcel Topilsky, Yosef Rodrig, A. Yellin, Ron N. Apte

**Affiliations:** 1 Departments of Pulmonary and Allergic Diseases Ichilov Hospital 6 Weizmann Street Tel-Aviv 64239 Israel; 2 Department of Human Microbiology Sackler Faculty of Medicine Tel-Aviv University Israel; 3 Clinical Chemistry Ichilov Hospital Tel-Aviv Sourasky Medical Center Israel; 4 Internal Medicine “H” Ichilov Hospital Tel-Aviv Sourasky Medical Center Israel; 5 Department of Thoracic Surgery Shiba Medical Centre Israel; 6 Department of Microbiology and Immunology Faculty of Health Sciences Ben Gurion University of the Negev Beer-Sheva Israel

## Abstract

Fibroblasts (Fb) from patients with sarcoidosis (SA) and hypersensitivity pneumonitis (HP) exhibited a lower proliferative capacity compared with Fb obtained from control (CO) and diffuse interstitial fibrosis patients (DIF). Proliferation of Fb from SA or lip patients was suppressed by autologous LPS-stimulated alveolar macrophages (AM) supernatants but not by those from CO patients. Similarly, alveolar macrophages (AM) derived supernatant, obtained from CO, did not suppress the proliferation of SA and HP Fb. AM from SA and HP patients secreted higher amounts of IL-1α and β compared with controls and compared with Fb from SA and HP patients. Steady levels of IL-1α and βmRNA were expressed in unstimulated and stimulated cultures. Fb from SA and HP patients could be stimulated by LPS to secrete significantly higher levels of PGE_2_ than those detected in supernatants from LPS stimulated Fb of DIF patients. Only the proliferation of Fb from SA and HP patients was sensitive to amounts of IL-1 equivalent to those detected in the lung of these diseases. As SA and HP are two diseases where irreversible deterioration occurs in only 20% of the patients, we hypothesize that mediators in the lung may modulate Fb proliferation. IL-1 of AM origin and PGE_2_ of Fb origin secreted at high levels, may be candidates for this suppression because it was abrogated by anti IL-1β and indomethacin.


Mediators of Inflammation 3, 445–452 (1994)

### Differential proliferative characteristics of alveolar fibroblasts in interstitial lung diseases: regulative role of IL-1 and $PGE_2$

Elizabeth Fireman,[1,CA] Shlomo Ben Efraim,[2] Joel Greif,[1] Hava Peretz,[3] Shmuel Kivity,[1] Marcel Topilsky,[4] Yosef Rodrig,[4] A. Yellin[5] and Ron N. Apte[6]

[1]Departments of Pulmonary and Allergic Diseases, Ichilov Hospital, 6 Weizmann Street, Tel-Aviv 64239, Israel; [2]Department of Human Microbiology, Sackler Faculty of Medicine, Tel-Aviv University, Israel; [3]Clinical Chemistry, and [4]Internal Medicine "H", Ichilov Hospital, Tel-Aviv Sourasky Medical Center, Israel; [5]Department of Thoracic Surgery, Shiba Medical Centre, Israel; [6]Department of Microbiology and Immunology, Faculty of Health Sciences, Ben Gurion University of the Negev, Beer-Sheva, Israel

[CA] Corresponding Author

**Fibroblasts (Fb) from patients with sarcoidosis (SA) and hypersensitivity pneumonitis (HP) exhibited a lower proliferative capacity compared with Fb obtained from control (CO) and diffuse interstitial fibrosis patients (DIF). Proliferation of Fb from SA or HP patients was suppressed by autologous LPS-stimulated alveolar macrophages (AM) supernatants but not by those from CO patients. Similarly, alveolar macrophages (AM) derived supernatant, obtained from CO, did not suppress the proliferation of SA and HP Fb. AM from SA and HP patients secreted higher amounts of IL-1α and β compared with controls and compared with Fb from SA and HP patients. Steady levels of IL-1α and βmRNA were expressed in unstimulated and stimulated cultures. Fb from SA and HP patients could be stimulated by LPS to secrete significantly higher levels of $PGE_2$ than those detected in supernatants from LPS stimulated Fb of DIF patients. Only the proliferation of Fb from SA and HP patients was sensitive to amounts of IL-1 equivalent to those detected in the lung of these diseases. As SA and HP are two diseases where irreversible deterioration occurs in only 20% of the patients, we hypothesize that mediators in the lung may modulate Fb proliferation. IL-1 of AM origin and $PGE_2$ of Fb origin secreted at high levels, may be candidates for this suppression because it was abrogated by anti IL-1β and indomethacin.**

**Key words:** Alveolar fibroblasts, Bronchoalveolar lavage, Interleukin-1, Interstitial lung diseases, Prostaglandin $E_2$

### Introduction

One of the most prominent histologic features of granulomatous and interstitial diseases in the lung is the close proximity of mononuclear cells with fibroblasts or the matrix secreted by them. Several studies characterized mononuclear cell-derived factors that stimulate[1,2] or inhibit[3,4] fibroblast growth and secretory functions *in vitro*. Other researchers have shown that cytokines are released spontaneously by macrophages isolated from lungs of animals exposed to a variety of stimulants, including cytotoxic drugs and mineral dusts,[5,6] as well as from patients with idiopathic pulmonary fibrosis (IPF)[7] and sarcoidosis (SA).[8] Furthermore, alveolar lining fluid collected by pulmonary lavage from patients with pulmonary fibrosis has been shown to contain fibrogenic cytokines, such as TGFβ[9] and TNFα,[10] pointing to the role of cytokines *in vivo*. These data suggest that cytokines are present *in situ* and may mediate the pathological manifestations of interstitial lung diseases. Lately, efforts have been directed towards the identification of macrophage-derived factors which affect fibroblast growth-function, while fewer studies were oriented towards the investigation of the immunoregulatory and pro-inflammatory role of fibroblasts. Recently, it was shown that murine fibroblasts, stimulated by cytokines and LPS, are able to generate IL-1α activity[11,12] and that rIL-1 and TNF stimulate normal adult human lung fibroblast to accumulate not to secrete IL-1β.[13] Moreover, other studies demonstrated that fibroblast strains secrete inflammatory mediators, such as prostaglandin $E_2$ ($PGE_2$), interleukin 6 (IL-6), interleukin 8 (IL-8), monocyte chemoattractant peptide (MCP-1) and colony-stimulating factor.[14–19] As we have previously shown that alveolar fibroblasts can be obtained from long-term cultures of bronchoalveolar cells recovered from patients with SA,[20] we decided to further characterize the interactions between alveolar macrophages and alveolar fibroblasts in interstitial lung diseases in an autologous system. Thus, in the present study we assessed the differential secretion of IL-1 and $PGE_2$ by these cells, in comparison with alveolar macrophages and the possible role of these mediators as suppressive agents of fibroplasia and fibrosis in these diseases.

© 1994 Rapid Communications of Oxford Ltd

### Patients and Methods

*Study population.* Eighteen patients, belonging to three groups were included in this study.

*Pulmonary sarcoidosis (SA).* Diagnosis was made in six untreated patients (three males and three females, mean age 37 ± 7 years), by clinical and roentgenological presentation, a positive Kveim test, or a positive biopsy of non-caseating granuloma. All patients were in Stage II sarcoidosis. None of them was a smoker.

*Diffuse interstitial fibrosis (DIF).* Three patients (two males and one female, mean age 61 ± 10 years). Diagnosis of DIF was made by roentgenological evidence of reticular infiltration and different degrees of interstitial fibrosis, demonstrated by transbronchial biopsy. None of them was a smoker.

*Hypersensitivity pneumonitis (HP).* Three patients belonged to this group (two males, one female, mean age 48 ± 6 years). Roentgenological evidence (X-rays and CT scan) showed reticular nodular pattern with predominant upper zone involvement. Bronchoalveolar lavage (BAL) analysis demonstrated features of a cell-mediated immune response with lymphocytosis. Lung histology was compatible with HP but no attempt was made to characterize the sensitizing antigens. None of them was a smoker.

*Control.* Six patients (three males and three females, mean age 44 ± 18 years) were admitted for investigation due to persistent cough. All of them presented chest roentgenograms within normal limits. None of them was a smoker.

None of the patients received any medicaments prior to the study. Written consent was obtained from each subject before bronchoscopy. Characterization of cell population present in BAL and pulmonary function test parameters of all patients examined are summarized in Table 1.

*Methods:*

*Bronchoalveolar lavage (BAL).* BAL was performed using a flexible fibre optic bronchoscope (BF-B2; Olympus Optical Co., Ltd, Tokyo, Japan) as previously described.[21] The cells were recovered by gentle aspiration. The percentage of lavage fluid (±SD) recovered from each group of patients was as follows: 67 ± 10% from CO and 58 ± 8% from ILD cases. The average total cells recovered was $5 \pm 1 \times 10^6$ cells from CO and $17 \pm 7 \times 10^6$ cells from ILD patients.

*Preparation of AM and AM supernatants.* AM were prepared as previously described.[21] Differential counts were performed on a Giemsa stained cytocentrifuge preparation (Cytospin; Shandon, Southern Products Ltd, Runcorn, Cheshire, UK), by counting a minimum of 500 cells. Cells were adjusted to a final concentration of $10^6$ cells/ml in RPMI 1640 medium, supplemented with 10% heat· inactivated FCS, 1% L-glutamine, and 1% streptomycin, penicillin, mycostatin complete medium (Biological Industries, Beit Haemek, Israel). The AM were purified by adherence in a 5% $CO_2$ humidified atmosphere for 1 h at 37°C. Identification of macrophages was done by morphology and nonspecific esterase staining and counted with an objective micrometer (Olympus Optical Co., Ltd, Tokyo, Japan). The adherent cell population contained more than 90% AM.

AM were cultured in 3-cm diameter plastic Petri dishes (Sterilin, Hounslow, Middlesex, UK) for either 24 h or 72 h. The 24 h period was found to be optimal for testing the production if IL-1 in the presence of lipopolysaccharide (LPS–*Escherichia coli* 055:B5; Difco Laboratories, Detroit, USA; 10 µg/ml) stimulated cultures. The 72 h period was chosen as the optimal time for release of $PGE_2$ in unstimulated cultures. Supernatants were recovered, filtered (Acrodisc 0.2 µ; Gelman Sciences) and stored at −70°C until determination for IL-1, and not longer than 2 weeks for $PGE_2$.

**Table 1.** BAL cells characteristics and pulmonary function tests (PFT) in all patients

| Patients[a] | Mac | Ly | Bas | Eos | Neut | DLCO | FVC | $FEV_1$ | $FEV_1$/FVC | TLC |
|---|---|---|---|---|---|---|---|---|---|---|
| SA (6) | | | | | | | | | | |
| Mean ± SD | 51 ± 15 | 48 ± 15* | 0.6 ± 0.4 | 0.5 ± 0.2 | 0.8 ± 0.6 | 99 ± 14** | 99 ± 9 | 87 ± 10 | 102 ± 6 | 97 ± 11 |
| HP (3) | | | | | | | | | | |
| Mean ± SD | 40 ± 8 | 58 ± 12* | 1.9 ± 1 | 0.9 ± 1.3 | 0.8 ± 0.4 | 114 ± 22** | 103 ± 14 | 104 ± 10 | 106 ± 3 | 103 ± 15 |
| DIF (3) | | | | | | | | | | |
| Mean ± SD | 70 ± 8 | 13 ± 13 | – | 8.4 ± 8*** | 12 ± 8*** | 69 ± 9 | 76 ± 8 | 77 ± 9 | 106 ± 6 | 86 ± 7 |
| CO (6) | | | | | | | | | | |
| Mean ± SD | 86 ± 5 | 13 ± 5 | – | – | – | 103 ± 12 | 99 ± 3 | 101 ± 12 | 109 ± 5 | 102 ± 5 |

*$p < 0.0001$ compared with CO group.
**$p < 0.001$ compared with DIF group.
***$p < 0.001$ compared with CO group.
Mac = macrophages; Ly = lymphocytes; Bas = basophils; Eos = eosinophils; Neut = neutrophils; DLCO = diffusion lung carbon oxide; FVC = forced vital capacity; $FEV_1$ = forced vital capacity second one; TLC = total lung capacity.
PFT were performed in all patients prior to the BAL.
Differential counts were performed by counting 500 cells of a Giemsa stain cytoprep as described in Patients and Methods.

*Preparation of alveolar fibroblasts.* The fibroblast line was derived from the bronchoalveolar cells as previously described.[20] First clones of proliferating fibroblasts appeared after 2–3 weeks of incubation in 3-cm diameter plastic Petri dishes (Sterilin, Hounslow, Middlesex, UK). After reaching confluence, usually 5–6 weeks later, the explant tissue was removed with trypsin-EDTA Type B (Biological Industries, Beit Haemek, Israel) for 10 min and cells were split 1:2 at confluency in 25 $cm^2$ tissue plastic culture flasks (Sterilin, Hounslow, Middlesex, UK). In all experiments the cells used were from passages 4–7.

*Preparation of pulmonary fibroblasts.* Stable lines of human pulmonary fibroblasts were used a control cells. Lung specimens from pneumonectomy specimens from patients with thoracic malignancies or benign lesions were minced into pieces of 2–4 mm and incubated in 1×5 cm Petri dishes (Sterilin, Hounslow, Middlesex, UK) containing 3 ml of complete DMEM. Every 72 h the non-adherent cells were removed by washing with PBS and fresh media was added. After 2 weeks, cultures reached confluence and the cells were split and used as described above.

*Preparation of Fb supernatants and Fb proliferation test.* Fb derived supernatants were obtained from 24 h LPS-stimulated Fb cultures cultured for 24 h with or without LPS. Aliquots of the supernatants were frozen at –70°C until used. Proliferation test was performed as previously described.[20] Briefly, 100 µl of Fbs suspended at $10^5$ Fb/ml were allowed to attach for 1–2 h. Aliquots (50 µl) of supernatants of LPS-stimulated AM were added. Proliferation was assessed after 72 h, and pulsed with 1 µCi $^3$H-Tdr (48 µCi/nmol specific activity, Rotem Industries Ltd, Beer-Sheva, Israel) for the last 16 h of culture. The proliferation of Fb in the presence of AM supernatants was compared with that of Fbs DMEM with a final concentration of 100 µg/ml LPS.

*Assay of prostaglandin and IL-1 production.* Aliquots of AM and Fb supernatants (24 h production) were assayed for $PGE_2$ by a radioimmunoassay (Advanced Magnetic Inc., MA, USA) and IL-1α and IL-1β production by a RIA Kit (Amerlex-M™ Magnetic separation, Amersham International PLC, Amersham, UK).

*Isolation of mRNA transcripts.* RNA was extracted by 100 µl of 4.0 M guanidium thiocyanate (GuSCN Sigma Chemical Co., St Louis, USA) from adherent $10^6$ cells/ml stimulated (10 µg LPS + 10 ng/ml IL-1α and β for 24 h and non-stimulated AFb. The mixture was overlaid on to 100 µl of 5.7 M CsCl and RNA was isolated after overnight ultracentrifugation at 35000 r.p.m. (Kontron Institute, Zurich, Switzerland) at 15°C. The pellet recovered by centrifugation was resuspended in 100 µl DEPC water, 300 µl pure ethanol and 30 µl 3.0 M sodium acetate. The RNA pellet (30′ at 15000 r.p.m.) was washed (100 µl 80% ethanol) and amplified using the reverse transcription-mix [RT mix: 3 µl 200 U/µl MMLV, moloney murine leukaemia virus-RT (BRL, Bethesda Research Laboratory, Gaithesburg, MD, USA), 1 µl 40 U/µl RNAasin, 6 µl 5XMMLV buffer, 3 µl random primers (Promega CA, Madison, USA), 3 µl of 1 mg/ml BSA (Sigma Chemicals Co., St Louis, USA) 1.5 µl of 10 mM dNTP mix-Pharmacia, Fine Chemicals AB, Uppsala, Sweden). Each sample contained 17.5 µl and the reaction was performed for 1 h at 42°C.

*PCR assay.* cDNA fragments were amplified (GeneAmp-Clontech Laboratories Inc., Palo Alto, CA, USA) in a polymerase chain reaction (PCR) mix containing 8 µl dNTPs mix 1.25 mM (Pharmacia Fine Chemicals AB, Uppsala, Sweden) and 0.25 µl *Taq* polymerase, 5 U/µl (Promega CA, Madison, USA) using thermal cycler cells (PT-100 MJ Research Inc., OSP, Cambridge MA, USA). β actin mRNA was evaluated concurrent with IL-1α and β mRNA as an internal control. Products of the combined reverse transcription-polymerase reaction (8 µl PCR product and 2 µl of gel loading buffer) were fractionated by electrophoresis in agarose stained with ethidium bromide and validated by matching predicted size 174/*Hae*III digest (Pharmacia Fine Chemicals AB, Uppsala, Sweden).

*Statistics.* Student's *t*-test was used for statistical evaluations using the Epistat Software, © 1984, T.L. Gustafson. The results are expressed as mean ± SD and values less than 0.05 were considered to be significant.

### Results

*Effect of AM supernatants on the proliferation of fibroblasts*: The basic proliferation rate of Fb from SA and HP patients was shown to be significantly lower than that of Fb in the CO group (Table 2).

AM-derived supernatants were tested for effects on the proliferation of Fb in an autologous culture set-up and in presence of AM supernatant of CO patients (Fig. 1) and on normal Fb lines (Fig. 2). The AM supernatants of SA and HP suppressed the prolifera-

**Table 2.** Proliferation rates of Fbs[a]

| Patient | SA-Fb | HP-Fb | CO-Fb |
|---|---|---|---|
| 1 | 7753 ± 1007 | 32677 ± 5257 | 62466 ± 5528 |
| 2 | 9399 ± 297 | 10509 ± 1256 | 28437 ± 2687 |
| 3 | 1801 ± 437 | 9182 ± 1609 | 25347 ± 5630 |
| 4 | 7613 ± 1007 | | 30271 ± 982 |
| 5 | 6047 ± 268 | | 46776 ± 2286 |
| 6 | 2633 ± 853 | | – |
| Mean ± SD | 5874 ± 2771* | 17459 ± 10776** | 38659 ± 14033 |

[a] Fibroblasts were cultured in complete DMEM as described in Patients and Methods. Thymydine uptake was measured after incubation of 72 h.
*$p<0.001$ compared with CO.
**$p<0.001$ compared with CO.

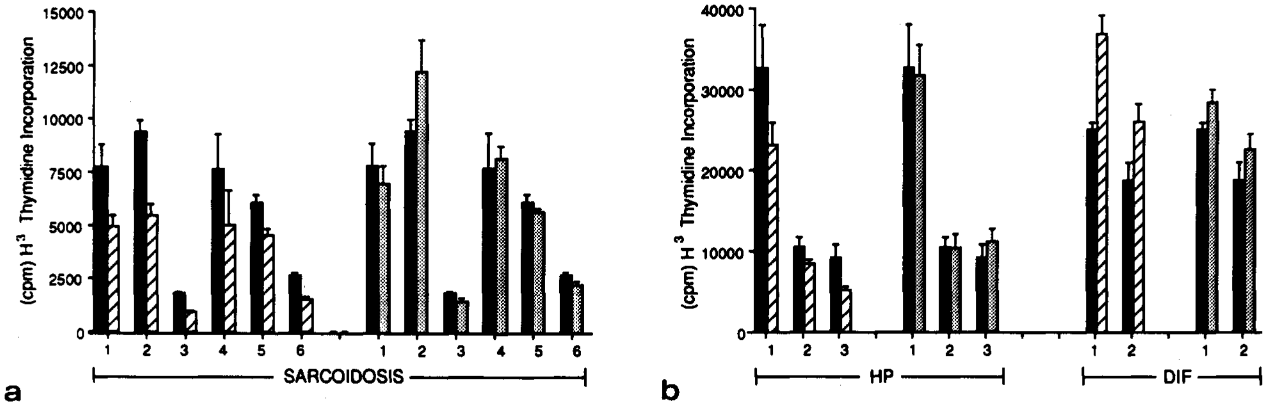

FIG 1. Effects of AM-derived supernatants on the proliferation of autologous Fb. Fb were incubated for 72 h in DMEM (2% FCS, antibiotics, antimycotic) in the presence or absence of autologous supernatants. (a) Six cases: ■ Fb incubated in DMEM; ▨ Fb incubated in DMEM + autologous AM supernatant of SA patients; ▩ Fb incubated in DMEM + AM supernatant of CO patients. (b) Left side, three HP cases; right, two DIF cases: ■ Fb incubated in DMEM; ▨ Fb incubated in DMEM + autologous AM supernatant of HP and DIF patients; ▩ Fb incubated in DMEM + AM supernatant of CO patients.

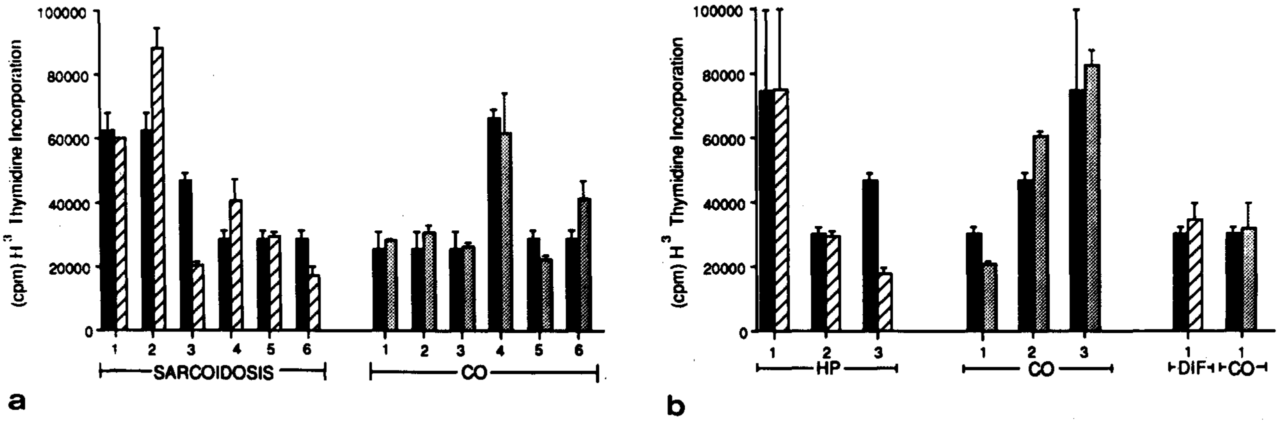

FIG. 2. Effects of AM-derived supernatants on the proliferation of control Fb. Fb were incubated for 72 h in DMEM (2% FCS, antibiotics, antimycotic) in the presence or absence of supernatants. (a) Six cases: ■ Fb incubated in DMEM; ▨ Fb incubated in DMEM + AM supernatants of SA patients; ▩ Fb incubated in DMEM + AM supernatants of CO patients. (b) From left to right: HP three cases; CO three cases; DIF and CO one case: ■ Fb incubated in DMEM; ▨ Fb incubated in DMEM + AM supernatants of HP and DIF; ▩ Fb incubated in DMEM + AM supernatants of patients.

tion of autologous Fb by 38 ± 7% (Fig. 1a, patients 1–6, left panel) and 31 ± 10% (Fig. 1b, patients 1–3, left panel), respectively. In contrast, CO AM-derived supernatants suppressed proliferation by only 13 ± 5% in four cases (Fig. 1a, patients 1, 3, 5 and 6, right panel) and enhanced by 30 ± 1% in two cases (Fig. 1a, patients 2 and 4, right panel) when tested on Fb of SA. When CO AM supernatants were tested on HP-Fb, 3% suppression in two cases (Fig. 1b, patients 1 and 2, right panel) and 20% enhancement in the third case ($p < 0.001$ between patients groups and controls). The SA-AM derived supernatants exerted a differential effect when tested on normal Fb: suppression of 34 ± 22% in three cases (Fig. 2a, patients 1, 3 and 6, left panel) and enhancement in three others 29 ± 18% (Fig. 2a, patients 2, 4 and 5, left panel). A similar pattern was seen in the HP group. Supernatants derived from DIF patients enhanced the proliferation of Fb in all cases tested.

Thus, in the autologous culture set-up, AM-derived supernatants from SA and HP patients exerted significant suppression on the proliferative capacity of alveolar Fb, which was much less pronounced when tested on normal Fb. In addition, AM-derived supernatants from control or DIF patients, did not exert a suppressive effect on Fb proliferation.

*Analysis of IL-1α, IL-1β and $PGE_2$ levels in AM and Fb supernatants*: LPS-induced supernatants from AM and Fb were tested for their ability to secrete IL-1α, β and $PGE_2$. As shown in Tables 3 and 4, the levels of IL-1α and β of stimulated SA and HP AM,

**Table 3.** IL-1α[a] levels in AM and Fb supernatants

| Diagnosis | AM | | Fb | |
|---|---|---|---|---|
| | –LPS | +LPS | –LPS | +LPS |
| SA (6) | 0.17 ± 0.03* | 0.78 ± 0.71* | 0.16 ± 0.05 | 0.17 ± 0.05 |
| HP (3) | 0.13 ± 0.0 4* | 0.5 ± 0.2** | 0.14 ± 0.05 | 0.2 ± 0.04 |
| DIF (3) | 0.08 ± 0.02 | 0.16 ± 0.02 | 0.14 ± 0.15 | 0.16 ± 0.01 |
| CO (6) | 0.05 ± 0.007 | 0.18 ± 0.04 | 0.18 ± 0.04 | 0.18 ± 0.02 |

[a] IL-1α was measured in Fb supernatants by RIA as described in Patients and Methods. Results are expressed as concentration of IL-1 (ng/ml).
*$p < 0.05$ compared with CO.
**$p < 0.001$ compared with CO.

**Table 4.** IL-1β[a] levels in AM and Fb supernatants

| Diagnosis | AM | | Fb | |
|---|---|---|---|---|
| | −LPS | +LPS | −LPS | +LPS |
| SA (6) | 0.09 ± 0.04** | 2.1 ± 2.0* | 0.14 ± 0.04 | 0.15 ± 0.04 |
| HP (3) | 0.08 ± 0.03** | 1 ± 0.6** | 0.13 ± 0.04 | 0.17 ± 0.04 |
| DIF (3) | 0.08 ± 0.02 | 0.16 ± 0.02 | 0.18 ± 0.06 | 0.17 ± 0.02 |
| CO (6) | 0.07 ± 0.02 | 0.17 ± 0.08 | 0.12 ± 0.04 | 0.12 ± 0.01 |

[a] IL-1β was measured in Fb supernatants by RIA as described in Patients and Methods. Results are expressed as concentration of IL-1 (ng/ml).
*$p < 0.05$ compared with CO and DIF.
**$p < 0.001$ compared with CO and DIF.

were significantly higher than those of CO cultures (2.1 ± 2.0 and 1 ± 0.6 ng/ml, compared with 0.17 ± 0.08 ng/ml for IL-1β, and 0.8 ± 0.7 and 0.5 ± 0.2 ng/ml compared with 0.2 ± 0.04 ng/ml for IL-1α, $p < 0.05$ compared with CO).

LPS did not affect the secretion if IL-1α and IL-1β and only baseline levels of those cytokines were produced by Fb irrelevant of their source. In contrast, when $PGE_2$ secretion was assessed (Table 5) it was demonstrated that LPS-stimulated Fb of SA and HP patients generated significantly higher levels of $PGE_2$ (0.36 ± 0.24 and 0.59 ± 0.27 ng/ml in stimulated supernatants of SA and HP patients compared with 0.16 ± 0.12 and 0.23 ± 0.19 ng/ml in unstimulated cultures). The high levels of $PGE_2$ secreted by stimulated Fb in SA and HP were significantly higher than those found in Fb recovered from DIF patients (0.36 ± 0.24 and 0.59 ± 0.27 ng/ml in SA and HP, compared with 0.06 ± 0.03 ng/ml in DIF, $p < 0.05$).

*Detection of IL-1α and IL-1β mRNA transcripts in Fb*: In order to assess whether the IL-1α and IL-1β genes are expressed in Fb of these diseases, we assessed the mRNA transcripts by PCR. IL-1α and IL-1β transcripts were constitutively found in stimulated, as well as non-stimulated, Fbs (Fig. 3a, b, c and d).

*Effects of exogenous IL-1β on Fb proliferation*: Exogenous IL-1β (concentrations in the range of 0.3–1000 ng/ml), were added to Fb cultures and proliferation was assessed by the tritiated thymidine incorporation. IL-1β, at concentrations of 0.35–62.5 ng/ml (Fig. 4a) and 0.35–125 ng/ml (Fig. 4b) significantly suppressed the basic proliferation rate of Fb by 46 ± 1.4% and 39 ± 0.9% ($p < 0.001$) compared with proliferation of Fb in complete medium (Fig. 4a and b). These concentrations include the range of IL-1β by AM of SA and HP patients (4–0.8 ng/ml). As a general trend in Fbs of the CO group, no suppressive activity was observed at minute concentrations of IL-1β (Fig. 4c). The modulatory effects of IL-1β were abrogated by anti-IL-1 antibodies or indomethacin (Table 6). A clear reversion of the suppression was achieved in the SA and HP group, but not in the CO and DIF groups.

### Discussion

AM are present within the alveoli and bronchioli while the fibrosis occurs in the interstitium. However, it is feasible to assume that AM affect the process of fibrosis because of their close proximity. The outcome of a number of interstitial lung diseases (ILD) results in severe pulmonary fibrosis. In contrast, patients with SA or HP, manifesting inflammatory or granulomatous diseases, usually heal without excessive scarring. In the present study, we assessed the fibroblast–macrophage interactions in ILD, in an attempt to understand the differential outcome of the diseases. We assessed the proliferative capacity of Fb and the secretion of cytokines/inflammatory products by AM and Fb, the latter being the target cells in these diseases.

We showed (Table 2) that Fb recovered from SA and HP, display significantly lower rates of background proliferation, compared with Fb recovered from normal tissue specimens even after serial passages for up to 2 months. The existence of Fb populations from fibrotic lungs which retain enhanced proliferative potential in culture, have already been reported[22,23] together with reports on decreased[24] or increased[25] cytokine production by fibroblasts in chronic GVHD (graft versus host dis-

**Table 5.** $PGE_2$[a] levels in AM and Fb supernatants

| Diagnosis | AM | | Fb | |
|---|---|---|---|---|
| | −LPS | +LPS | −LPS | +LPS |
| SA (6) | 0.06 ± 0.02 | 0.31 ± 0.3 | 0.16 ± 0.12* | 0.36 ± 0.24*** |
| HP (3) | 0.09 ± 0.02 | 0.27 ± 0.14 | 0.23 ± 0.19 | 0.59 ± 0.27 |
| DIF (3) | 0.5 ± 0.3 | 1.2 ± 0.5**** | 0.6 ± 0.03 | 0.06 ± 0.03** |
| CO (6) | 0.04 ± 0.01 | 0.41 ± 0.31 | 0.49 ± 0.20 | 0.46 ± 0.20 |

[a] $PGE_2$ was measured in Fb supernatants by RIA as described in Patients and Methods. $PGE_2$ levels are expressed in ng/ml.
*$p < 0.05$ compared with CO.
**$p < 0.01$ compared with CO.
***$p < 0.05$ compared with DIF.
****$p < 0.02$ compared with CO, SA and HP.

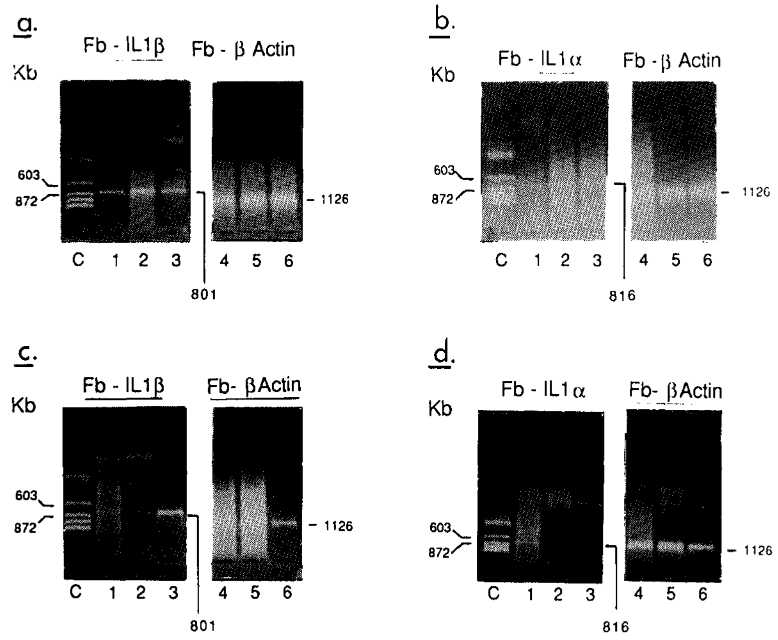

FIG. 3. Detection of IL-1α and IL-1β mRNA transcripts in Fb. mRNA was isolated as described in Patients and Methods and reverse transcripted in a RT reaction. cDNA fragments were amplified in a PCR reaction. (a,b) IL-1α and β transcripts of Fb in SA; (c,d) IL-1α and β transcripts of Fb in CO. Each experiment is compared with positive transcription of β actin.

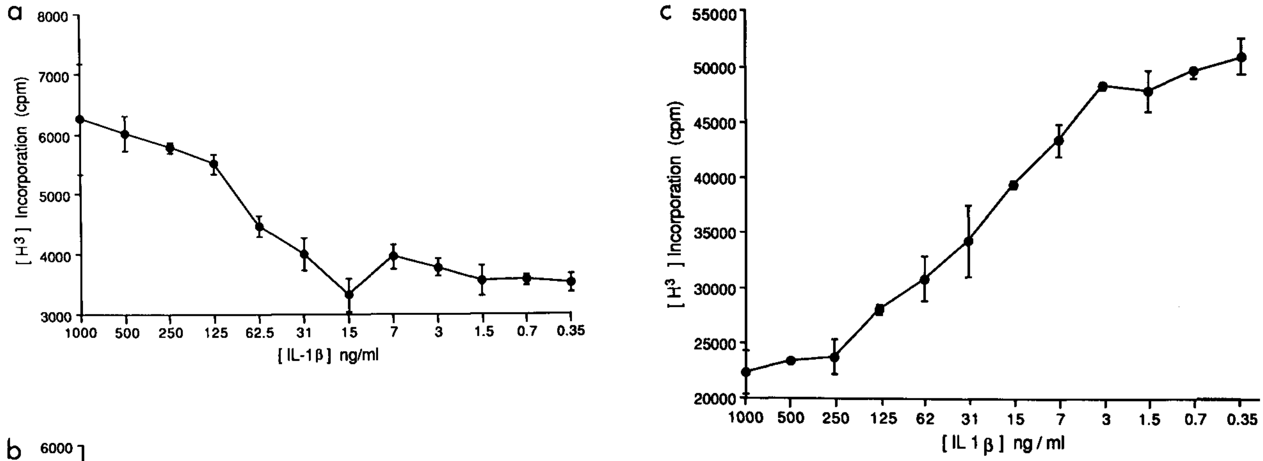

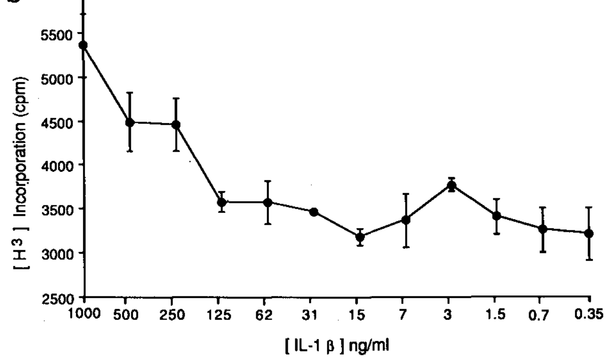

FIG. 4. Effect of exogenous IL-β on the proliferation of Fb. (a) SA Fb (basic proliferation of Fb = 6600 ± 545 cpm). (b) HP Fb (basic proliferation of Fb = 5429 ± 345 cpm). (c) CO Fb (basic proliferation of Fb = 54173 ± 1327 cpm).

**Table 6.** Effect of indomethacin and anti-IL-1β antibodies on IL-1β induced suppression on Fb proliferation[a]

| | SA | HP | DIF | CO |
|---|---|---|---|---|
| Medium | 4478 ± 235 | 5430 ± 343 | 15726 ± 150 | 54173 ± 1327 |
| IL-1β (1.5 ng/ml) | 3561 ± 257 | 3404 ± 8 | 12500 ± 500 | 47808 ± 1866 |
| IL-1β (1.5 ng/ml) + Ind (1 μg/ml) | 7516 ± 354 | 5062 ± 352 | 8306 ± 914 | 48977 ± 9649 |
| IL-1β (1.5 ng/ml) + anti-IL-1 (20 μg/ml) | 5508 ± 142 | 10782 ± 1052 | 2648 ± 108 | 46689 ± 780 |

[a] cpm of $^3$[H] thymidine incorporation of fibroblasts. Indomethacin and anti-IL-1 were added and cells were incubated for 72 h. The experiment was repeated three times with similar results.

ease) and rheumatoid arthritis, respectively. The possibility that alveolar Fb differed from control Fb in the method of recovery seems unlikely, as it was shown by Davis *et al.*[26] that alveolar lavage fibroblasts displayed similar distributions of interdivision time as in foetal and adult lung tissue fibroblasts.

We showed also that AM-derived supernatants suppressed the proliferation of autologous Fb, recovered from patients with SA and HP, but not of Fb from DIF patients or CO (Fig. 1a and b). However, as described also by others,[27] AM supernatants from SA patients, when tested on normal lung fibroblasts, either inhibit or enhance their proliferation, and no clear pattern of response could be concluded. These results may indicate that our autologous culture set-up may approximate the *in vivo* situation.

As SA and HP are two diseases where irreversible deterioration in pulmonary function occurs in only 20% of patients, we hypothesize that inflammatory mediators secreted by AM or Fb may mediate the suppression in the proliferative capacity of autologous Fb in SA and HP. We found here and in previous studies[21,28] that IL-1 is actively secreted from AM in SA and HP patients. Thus, the secretion of IL-1 together with secretion of TNFα,[29–31] which may possibly mediate the suppression of Fb proliferation in these diseases. This observation was also confirmed by Elias *et al.*[3,4,32]

In order to investigate the role of IL-1 in the Fb suppression we examined first whether exogenous stimuli can potentiate Fb to generate soluble IL-1α and β. We could detect IL-1α and IL-1β gene transcription in CO or LPS/IL-1 stimulated Fb from CO and SA patients. Subsequently, we found that stimulated and non-stimulated Fb secrete low detectable levels of both species of IL-1 (0.12–0.17 ng/ml). The same stimulus induced AM to produce high levels of IL-1α and β. These results are in agreement with previous findings[33,34] demonstrating that stimulated Fb contain more (1–10%) IL-1 mRNA than LPS-stimulated monocytes, but they produce less detectable IL-1β (<0.04%). The mechanism of IL-1 secretion is still obscure, as it lacks a signal peptide. A role for plasmin or plasmin-like proteases for the release of IL-1 has been reported in macrophages.[35] It may be that this enzymatic activity is lacking in Fb. This dichotomy between IL-1 gene transcription and expression of biological activity has already been demonstrated in stimulated macrophages and Fb[36,37] and may suggest that IL-1 activity may be regulated at multiple levels, such as transcription, stability of mRNA, translation and post-translational events. In our case, we postulate that one of these regulatory post-transcriptional events may be mediated by prostaglandins, as this mechanism was already demonstrated for normal AM to suppress cytokine production.[38] In fact, we showed that only fibroblasts recovered from SA and HP patients may be induced to release significant amounts of $PGE_2$ upon stimulation with LPs.

Two points have to be considered concerning the possible involvement of IL-1 in suppressing the proliferative capacity of lung Fb: (i) Fb from SA and HP patients *in vivo* are exposed to high levels of IL-1 of AM origin; (ii) the exogenous effect of this cytokine may be differential on quiescent versus rapidly proliferating fibroblasts. In fact, we tested the effects of exogenous IL-1β on the proliferation of normal Fb, as well as Fb cells obtained from SA and HP patients.

Our results indicate that IL-1β, at the levels equivalent to those present in the microenvironment in the lungs of SA and HP patients (0–4 ng/ml), exerts a suppressive effect on slowly subconfluent proliferating alveolar Fb from these diseases, whereas at levels detected in normal lungs (0–0.2 ng/ml), enhance the replication of actively proliferating Fb recovered from control lungs (Fig. 3a–c). Similar dichotomic effects have already been demonstrated for the regulation of fibroblast proliferation by recombinant interferons σ, α and β.[8] Indeed, using Fb, as well as other cell types, contrasting effects of IL-1 on cell proliferation were reported.[39–43] This may result from differential sensitivity of various cells to the direct mitogenic effects of IL-1 or to a distinct cytokine cascade induced by IL-1. In our case, it may be possible that IL-1 induces in SA and HP, Fb suppressive cytokines (such as TGF-β, IL-6 and possibly IL-10) or other mediators (such as $PGE_2$).

In conclusion, we postulate that the benign course of the disease of most patients with SA and HP involves a downregulation in the proliferative capacity and possibly alters the secretory function of Fb.

### References

1. Bitterman PB, Rennard SI, Hunnighake GW, Crystal RG. Human alveolar macrophage growth factor for fibroblasts; regulation and partial characterization. *J Clin Invest* 1982; **70:** 806–822.
2. Leslie CC, Musson RA, Henson PM. Production of growth factor activity for fibroblasts by human monocyte derived macrophages. *J Leuk Biol* 1984; **36:** 143–159.
3. Elias JA, Rossman MD, Zurier RB. Human alveolar macrophage inhibition of lung fibroblast growth: a prostaglandin-dependent process. *Am Rev Respir Dis* 1985; **131:** 94–99.
4. Elias JA. Tumor necrosis factor interacts via Elias, interleukin-1 and interferons to inhibit fibroblast proliferation via fibroblast prostaglandin-dependent and independent mechanism. *Am Rev Respir Dis* 1988; **138:** 652–658.
5. Bauman MD, Jetten AM, Brody AR. Biologic and biochemical characterization of a macrophage derived growth factor for rat lung fibroblasts. *Chest* 1987; **91:** 15S–16S.
6. Schmidt JA, Oliver CN, Lepe-Zuniga JL, Green I, Gery I. Silica-stimulated monocytes release fibroblast proliferation factor identical to interleukin-1. *J Clin Invest* 1984; **73:** 1462–1472.
7. Martinet Y, Bitterman PB, Mornex JF, Grotensdorst GR, Martin GR, Crystal RG. Activated human monocytes express the c-sis proto-oncogene and release a mediator showing PDGF-like activity. *Nature* 1986; **319:** 158–160.
8. Lamontagne L, Gauldie J, Stadnyk A, Richards C, Jenkins E. *In vivo* initiation of unstimulated *in vitro* interleukin-1 released by alveolar macrophages. *Am Rev Respir Dis* 1985; **131:** 326–330.
9. Khallil N, Bereznay O, Sporn M, Grennberg AH, Macrophage production of transforming growth factor β and fibroblast collagen synthesis in chronic pulmonary inflammation. *J Exp Med* 1989; **170:** 727–737.
10. Piguet PF, Collart MA, Grau GE, Kapanci Y, Vasalli P. Tumor necrosis factor/cachectin plays a key role in bleomycin induced pneumopathy and fibrosis. *J Exp Med* 1989; **170:** 655–663.
11. Huliel M, Douvdevani A, Segal S, Apte RN. Regulation of interleukin 1 generation in immune-activated fibroblasts. *Eur J Immunol* 1990; **20:** 731–738.
12. Huliel M, Douvdevani A, Segal S, Apte RN. Different regulatory levels involved in the generation of hemopoietic cytokines (CSFs and IL-6) in fibroblasts stimulated by inflammatory products. *Cytokine* 1993; **5:** 47–56.
13. Elias JA, Reynolds MM, Kotloff RM, Kern JA. Fibroblast interleukin 1β: synergistic stimulation by recombinant interleukin 1 and tumor necrosis factor and posttranscriptional regulation. *Proc Natl Acad Sci* 1989; **86:** 6171–6175.
14. Elias JA, Gustilo, Baeder W, Freudlich B. Synergistic stimulation of fibroblast prostaglandin production by recombinant interleukin-1 and tumor necrosis factor. *J Immunol* 1987; **138:** 3812–3816.
15. Le J, Weinstein D, Gubler V, Vicek J. Induction of membrane associated interleukin-1 by tumor necrosis factor in human fibroblasts. *J Immunol* 1987; **138:** 2137–2142.
16. Laeson CG, Zacharie CO, Oppenheim JJ, Matsushima K. Production of monocyte chemotactic and activating factor (MCAF), by human dermal fibroblasts in response to interleukin 1 or tumor necrosis factor. *Biochem Biophys Res Commun* 1989; **160:** 1403–1408.
17. Yoshimura T, Leonard JEJ. Secretion of human fibroblasts of monocyte chemoattractant protein-1, the gene product of gene JE. *J Immunol* 1990; **144:** 2377–2383.
18. Rollins BJ, Stier P, Ernst T, Wong CG. The human homolog of the JE gene encodes a monocyte secretory protein. *Mol Cell Biol* 1989; **9:** 4687–4695.
19. Rolfe MW, Kunkel SL, Standiford TJ, Censue SW, Allen RM, Evanoff HL, Phan SH, Streiter RM. Pulmonary fibroblast expression of IL-8: a model for alveolar macrophage-derived cytokine networking. *Am J Respir Cell Mol Biol* 1991; **5:** 493–501.
20. Fireman E, Ben Efraim S, Messer G, Dabush S, Greif J, Topilsky M. Cell-free supernatants of sarcoid alveolar macrophages suppress proliferation of sarcoid alveolar fibroblast. *Clin Immunol Immunopathol* 1991; **59:** 368–378.
21. Fireman E, Ben Efraim S, Greif J, Alguetti A, Ayalon D, Topilsky M. Suppressive activity of alveolar macrophages and blood monocytes from interstitial lung diseases: role of released soluble factors. *Int J Immunopharmacol* 1989; **7:** 751–760.
22. Jordana M, Schulman J, McSharry C, Irving LB, Newhouse MT, Jordana G, Gauldie J. Heterogenous proliferative characteristics of human adult lung fibroblast lines and clonally derived fibroblasts from control and fibrotic tissue. *Am Rev Respir Dis* 1988; **137;** 579–584.
23. Raghu G, Chen Y, Rusch V, Rabinovitch PS. Differential proliferation of fibroblasts cultured from normal and fibrotic human lungs. *Am Rev Respir Dis* 1988; **138:** 703–708.
24. Mekori YA, Huleihel M, Baram D, Apte RN. Depressed IL-1 production by chronic GVHD dermal fibroblasts. *Eur Cytokine Net* 1990; **2:** 77–83.
25. Bucala C, Ritchlin C, Winchester R, Cerami A. Constitutive production of inflammatory and mitogenic cytokines by rheumatoid synovial fibroblasts. *J Exp Med* 1991; **173:** 569–574.
26. Davis GS, Moehring JM, Absher PM, Brody AR, Kelly J, Low RB, Green GM. Isolation and characterization of fibroblasts obtained by pulmonary lavage of human subjects. *In vitro* 1979; **15:** 612–623.
27. Elias JA, Rossman MD, Daniele RP. Blood and lung mononuclear cell inhibition of fibroblast growth in sarcoidosis. *Am Rev Respir Dis* 1984; **130:** 1050–1058.
28. Hunnighake GW. Release of interleukin-1 by alveolar macrophages of patients with active pulmonary sarcoidosis. *Am Rev Respir Dis* 1984; **129:** 569–572.
29. Strasz J, Mannel DN, Pfeofer S, Bokowski A, Ferlinz F, Quernheim JM. Spontaneous monokine release by alveolar macrophages in chronic sarcoidosis. *Int Arch Allergy Appl Immunol* 1991; **96:** 68–75.
30. Fireman E, Aderka D, Ben Efraim S, Greif J, Wallach D, Topilsky M. Suppressive effect of TNFα and IL-1 on alveolar fibroblast proliferation in sarcoidosis. *Mediators of Inflammation* 1992; **1:** 235–240.
31. Denis M, Cournier Y, Fournier M, Tardif J, Laviolette M. Tumor necrosis factor plays an essential role in determining hypersensitivity pneumonitis in mouse model. *Am J Respir Cell Mol Biol* 1991; **5:** 477–483.
32. Elias JA, Freundlich BB, Kern JA, Rosenbloom J. Cytokine networks in the regulation of inflammation and fibrosis in the lung. *Chest* 1990; **97:** 1439–1445.
33. Rochemonteix-Galve B, Dayer JM, Junod AF. Fibroblast-alveolar cell interactions in sarcoidosis and idiopathic pulmonary fibrosis; evidence for stimulatory and inhibitory cytokine production by alveolar cells. *Eur Respir J* 1990; **3:** 653–664.
34. Kern JA, Lamb RJ, Reed JC, Daniele RP, Nowell PC. Dexamethasone inhibition of interleukin-1β production of human monocyte: posttranscriptional mechanisms. *J Clin Invest* 1988; **81:** 237–244.
35. Matsushima K, Taguchi M, Kovacs EJ, Young HA, Oppenheim JJ. Intracellular localization of human monocyte associated interleukin-1 activity and release of biologically active IL-1 from monocytes by trypsin and plasmin. *J Immunol* 1986; **136:** 2883–2891.
36. Dinarello CA, Wolff SK. Mechanism of disease: the role of interleukin 1 in disease. *N Engl J Med* 1992; **328:** 106–113.
37. Douvdevani A, Mahmud M, Segal HS, Apte RN. Aberrations in interleukin-1 expression in oncogene-transformed fibrosarcoma lines: constitutive interleukin-1α transcription and manifestation of biological activity. *Eur Cytokine Net* 1991; **2:** 257–264.
38. Kundsen PJ, Dinarello CA, Strom TB. Prostaglandin posttranscriptionally inhibit monocyte expression of interleukin-1 activity by increasing intracellular cyclic adenosine monophosphate. *J Immunol* 1986; **137:** 3189–3194.
39. Schmidt JA, Mizel SB, Cohen D, Green I. Interleukin 1, a potential regulator of fibroblast proliferation. *J Immunol* 1982; **128:** 2177–2182.
40. Rosenwasser LJ, Dinarello CA. Ability of human leukocytic pyrogen to enhance phytohemaglutinin-induced murine thymocyte proliferation. *Cell Immunol* 1981; **63:** 134–142.
41. Libby P, Friedman GB, Salomon RN. Cytokines as modulators of cell proliferation in fibrotic diseases. *Am Rev Respir Dis* 1989; **140:** 1114–1117.
42. Fujiwara H, Kleinhenz ME, Wallis RS, Ellner JJ. Increased interleukin-1 production and monocyte suppressor cell activity associated with human tuberculosis. *Am Rev Respir Dis* 1986; **133:** 73–77.
43. Floege J, Topley N, Hoppe J, Barret TB, Resch K. Mitogenic effect of platelet-derived growth factor in human glomerular mesengial cells: modulation and/or suppression by inflammatory cytokines. *Clin Exp Immunol* 1991; **86:** 334–341.

**Received 16 June 1994;**
**accepted in revised form 5 August 1994**
